# Sex differences in recovery from postoperative sarcopenia during adjuvant CAPOX therapy for colorectal cancer

**DOI:** 10.1007/s00432-024-06013-9

**Published:** 2024-10-26

**Authors:** Hiroaki Nozawa, Shinya Abe, Kentaro Abe, Yumi Yokota, Shunsuke Hori, Mitsutaka Yakabe, Kazuhito Sasaki, Shigenobu Emoto, Yuichiro Yokoyama, Hirofumi Sonoda, Koji Murono, Hiroyuki Matsuzaki, Yuzo Nagai, Takahide Shinagawa, Masahiro Akishita, Soichiro Ishihara

**Affiliations:** 1 Department of Surgical Oncology, The University of Tokyo, 7-3-1 Hongo, Bunkyo-ku, Tokyo, 113-8655 Japan; 2 Department of Geriatric Medicine, The University of Tokyo, Tokyo, Japan; 3 Tokyo Metropolitan Institute for Geriatrics and Gerontology, Tokyo, Japan

**Keywords:** Body composition, Sex difference, Adjuvant chemotherapy, CAPOX, Colorectal cancer

## Abstract

**Background:**

Women are predisposed to develop intolerance to cancer chemotherapy. Sarcopenia and chemotherapy are mutually related. Women are generally intolerable to chemotherapeutics such as 5-fluorouracil. Although adjuvant oxaliplatin-based chemotherapy, e.g. CAPOX is commonly used to treat colorectal cancer, its effects on patients in terms of sarcopenia and sex remain unknown. We investigated sex disparities in the impacts of CAPOX on body composition in this study.

**Methods:**

We conducted a prospective study on diagnostic metrics used for sarcopenia in colorectal cancer patients receiving adjuvant CAPOX. Evaluations of the nutritional status by the Mini-Nutritional Assessment (MNA), gait speed, grip strength, skeletal muscle mass, fat mass, and bone mineral content using a body composition analyzer were performed in the first, fourth, and eighth cycles of CAPOX (first, second, and third measurements, respectively).

**Results:**

Among 80 eligible patients, 61 completed four CAPOX cycles. The median differences in MNA, gait, grip strength, muscle mass, fat mass, and bone mineral content between the first and second measurements for men (*n* = 35) and women (*n* = 26) were + 10.5% and + 2.9% (*p* = 0.067), + 4.5% and − 2.6% (*p* = 0.16), + 1.8% and + 2.8% (*p* = 0.66), + 2.7% and + 1.3% (*p* = 0.021), + 4.5% and + 3.5% (*p* = 0.59), and + 3.3% and + 0.0% (*p* = 0.006), There were no sex differences in comparisons of the above metrics between the first and third measurements in 34 patients who completed eight CAPOX cycles (19 wen and 15 women).

**Conclusions:**

Early cycles of adjuvant CAPOX may have a negative impact on the postoperative recovery of several metrics for diagnosing sarcopenia in women.

**Supplementary Information:**

The online version contains supplementary material available at 10.1007/s00432-024-06013-9.

## Introduction

With recent advances in genetic and molecular testing as well as chemotherapeutic drugs, systemic therapy is now recommended for more patients with colorectal cancer (CRC) for not only palliative purposes, but also in the adjuvant setting (Dekker et al. 2019). Accumulating evidence suggests a sex bias in chemotherapy-related toxicity. Post-hoc analyses of randomized clinical trials (RCTs) on CRC demonstrated that palliative or adjuvant 5-fluorouracil (5-FU) monotherapy caused more frequent, more severe, and broader toxicities in female patients than in male patients (Sloan et al. 2002; Chansky et al. 2005). Another pooled analysis of five RCTs on metastatic CRC indicated greater toxicities with 5-FU and oxaliplatin therapy in women (Abdel-Rahman 2019). Even in the adjuvant setting, an analysis of the ACCENT database, which includes 34,640 patients from 27 RCTs, showed greater toxicities in women treated with 5-FU and oxaliplatin or irinotecan therapy (Wagner et al. 2021; De Francia et al. 2022). We also reported that women were at a higher risk of early dose-limiting toxicities (DLTs), namely, chemotherapy-induced toxicities of grade 2 by the Common Terminology Criteria for Adverse Events (CTCAE) (National Cancer Institute 2017), dose reductions, and unscheduled treatment delays in adjuvant CAPOX therapy after the resection of CRC (Nozawa et al. 2021). We found that sarcopenia was predominant in women and was associated with early DLTs (Nozawa et al. 2021).

Sarcopenia, a condition characterized by the loss of skeletal muscle mass, strength, and function, may develop with cancer or as a consequence of receiving cancer treatment (Cruz-Jentoft et al. 2010). Among many aspects of sarcopenia, body composition, including skeletal muscle mass, may be easily evaluated. Accumulating evidence suggests that body composition may have an impact on the tolerability of 5-FU and other cytotoxic drugs in patients with various malignancies, including CRC (Prado et al. 2007; William et al. 2018). On the other hand, many studies suggested that cytotoxic chemotherapy may induce the loss of skeletal muscle, adipose tissue, and bone mineral density in metastatic CRC (Blauwhoff-Bsukemolen et al. 2016; Dolly et al. 2020). Moreover, the loss of muscle mass caused by chemotherapy was reportedly predictive of poor survival in patients with metastatic CRC (Blauwhoff-Bsukemolen et al. 2016; Miyamoto et al. 2015). In the palliative setting, cancer disease progression itself may mainly contribute to prominent muscle wasting (Tisdale 2009). However, in the CAIRO-3 study on metastatic CRC, a subpopulation of patients recovered from sarcopenia induced by doublet therapies during the observation or maintenance period (Kurk et al. 2018), suggesting the definitive impact of chemotherapy on skeletal muscle mass and related functions. Consistent with these findings, adjuvant chemotherapy was shown to decrease skeletal muscle mass in patients after the complete removal of gastrointestinal cancer (Yamaoka et al. 2014; Oflazoglu et al. 2020).

Regarding the relationship between sarcopenia and chemotherapy-related toxicity, a retrospective study using data from the CAIRO-3 trial showed that sarcopenia and/or muscle loss was associated with an increased risk of DLTs in palliative systemic therapy (Kurk et al. 2019). Similarly, the C-SCANS study demonstrated that a lower muscle mass evaluated on computed tomography (CT) scans was associated with more severe adverse events and poor adherence in patients receiving adjuvant FOLFOX (Cespedes Feliciano et al. 2017). In another study, a low psoas muscle area on CT scans was related with toxicities caused by adjuvant FOLFOX and a poor prognosis in stage III colon cancer patients (Jung et al. 2015). Therefore, body composition and chemotherapy tolerability mutually affect each other.

Although skeletal muscle mass is considered to be fundamental to a clinical diagnosis of sarcopenia, the European Working Group on Sarcopenia in Older People proposed a diagnostic algorithm for sarcopenia in research and practice, in which muscle mass and strength as well as physical performance are cardinal requirements (Cruz-Jentoft et al. 2010). Due to anthropometric and cultural differences from Western countries, the Asian Working Group for Sarcopenia additionally proposed screening to identify Asian patients at risk of developing sarcopenia using questionnaires or calf circumferences (Chen et al. 2020).

Limited information is available on sex differences in the changes induced in body components by chemotherapy. Antoun et al. found female-specific decreases in skeletal muscle density and subcutaneous adipose tissue by the XELIRI or FOLFIRI regimen for metastatic CRC (Antoun et al. 2019). However, the mechanisms by which adjuvant chemotherapy affects body composition and whether it differs between men and women remain unclear. Many guidelines currently recommend regimens that include oxaliplatin as adjuvant chemotherapy for high-risk stage II and stage III CRC (Hashiguchi et al. 2020; Argilés et al. 2020; Yoshino et al. 2021; National Comprehensive Cancer Network 2024a, b). Therefore, we herein performed a prospective study to investigate how metrics for diagnosing sarcopenia change during adjuvant chemotherapy including oxaliplatin and examined sex disparities in these parameters.

## Materials and methods

### Study cohort

In this prospective observational study, which was approved by the Ethics Committee of the University of Tokyo (Reference No:2021363NI), we started to recruit patients who received adjuvant CAPOX therapy after curative resection for primary CRC in March 2022. We excluded patients who received neoadjuvant chemotherapy and/or chemoradiation therapy. Moreover, the diagnostic metrics of sarcopenia were analyzed only among patients who completed scheduled cycles of adjuvant chemotherapy in order to minimize the effects of its discontinuation.

Patients were followed-up at our hospital or affiliated institutions until May 31, 2024 or death, whichever came first.

### Adjuvant CAPOX therapy

We recommend oxaliplatin-based adjuvant chemotherapy, e.g. CAPOX, to high-risk stage II and stage III CRC patients at our hospital, according to the latest guidelines of the Japanese Society for Cancer of the Colon and Rectum (Hashiguchi et al. 2020). CAPOX consisted of the intravenous infusion of 130 mg/m^2^ oxaliplatin and the oral administration of capecitabine at a dose of 1,000 mg/m^2^ twice daily for two weeks. The treatment course was repeated every three weeks (Schmoll et al. 2015). Eight-cycle CAPOX therapy was scheduled. However, based on the findings of the IDEA collaboration (Grothey et al. 2018; André et al. 2020), short-course CAPOX (four cycles) was allowed for patients with stage II or low-risk stage III CRC as well as those with frailty, other systemic conditions, or the elderly after shared decision making. Adverse events were graded by CTCAE v5.0 (National Cancer Institute 2017).

### Evaluation schedule

**Fig. 1:**
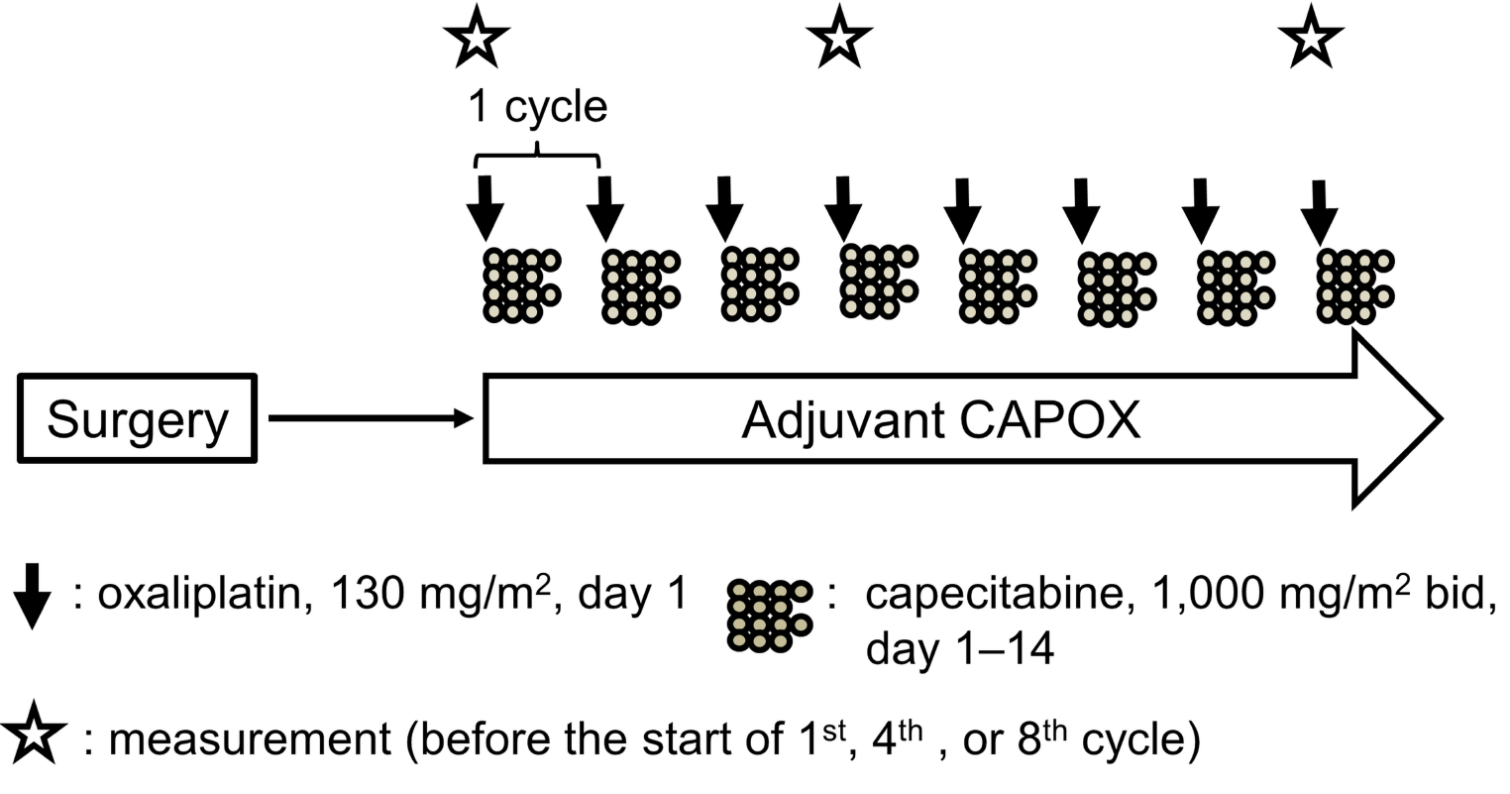
Schematic schedule of measurements during adjuvant CAPOX. The standard starting doses of oxaliplatin and capecitabine are shown

An evaluation comprising the measurement domains described below was conducted prior to the initiation of the first cycle of adjuvant CAPOX therapy (‘first measurement’). Measurements were again performed at the fourth and eighth cycles of CAPOX (‘second measurement’ and ‘third measurement’, respectively), as illustrated in Fig. 1. We also confirmed that no patients were under conditions such as receiving intravenous infusions or edema, which may affect resistance and reactance in a bioelectrical impedance analysis (BIA) as mentioned below. However, there were no dietary or urinary restrictions before each measurement.

### Nutritional assessment

We utilized the Mini-Nutritional Assessment (MNA), a validated evaluation form for screening sarcopenia, which comprises 18 questionnaires under four different domains, namely, anthropometric, general, dietary, and subjective/psychological (Guigoz 2006). The total score ranges between 0 and 30; a score of 24 or higher indicates a good nutritional status. A score between 17 and 23.5 implies a risk of malnutrition, whereas protein-calorie malnutrition may occur when the score is below 17 (Guigoz 2006).

### Gait speed

Usual gait speed may be used as a parameter for physical performance. In the present study, gait speed was calculated as the time to walk 6 m during a 10-m trial. Speed was shown as the mean of two walking trials.

### Grip strength

Grip strength, one of the methods employed to assess muscle strength, was measured using a digital hand dynamometer (EKJ077, EVERNEW Inc., Tokyo, Japan). The grip size was adjusted for each participant. Participants stood upright with the shoulder adducted and both arms extended downward a little away from the body. They squeezed the hand dynamometer with as much force as possible with verbal encouragement. They repeated the test two times for each hand, alternating sides with an interval of at least 15 s. The mean grip strength of all trials in kg was calculated.

### Body composition assessment

For body composition assessment, BIA was used as a non-invasive indirect method despite of its several disadvantages, because other methods such as CT scans and dual-energy X-ray absorptiometry are complex, expensive and time-consuming, or potentially harmful due to radiation exposure (Kyle et al. 2004). Body composition was measured in the standing position using a commercial octapolar BIA device (InBody770; InBody Japan. Inc., Tokyo, Japan). Patients took the retractable handles and held it firmly with their arms extended around a 30° angle to the trunk. The measurement was not repeated within a unit. Estimates of total skeletal muscle mass, fat mass, and bone mineral content were calculated using the data management software, Lookin’Body120 (InBody Japan. Inc., Tokyo, Japan), connected to the BIA device.

### Collection of other data

The following clinicopathological variables were retrieved from our prospectively collected database: sex, age, body mass index, serum levels of albumin and hemoglobin before the first CAPOX cycle, the Eastern Cooperative Oncology Group Performance Status, comorbidities, the primary tumor location and histology, and the TNM pathological classification of tumors at diagnosis according to the American Joint Committee on Cancer staging manual (Brierley et al. 2016). In the case of multiple CRC, the most advanced T and N stages were recorded. The diagnosis of sarcopenia was made at each measurement using the consensus of the Asian Working Groups for Sarcopenia (Chen et al. 2020).

### Statistical analysis

Qualitative variables were compared using Fisher’s exact test or the *χ*^2^ test with Yates correction where appropriate. Continuous variables were tested by the Wilcoxon rank-sum test or Kruskal-Wallis test. Differences were considered to be significant at a *p-*value less than 0.05. JMP statistical software version 17.2.0 (SAS Institute, Cary, NC, USA) was used for all statistical analyses.

## Results

### Selection and clinicopathological features of enrolled patients

**Fig. 2:**
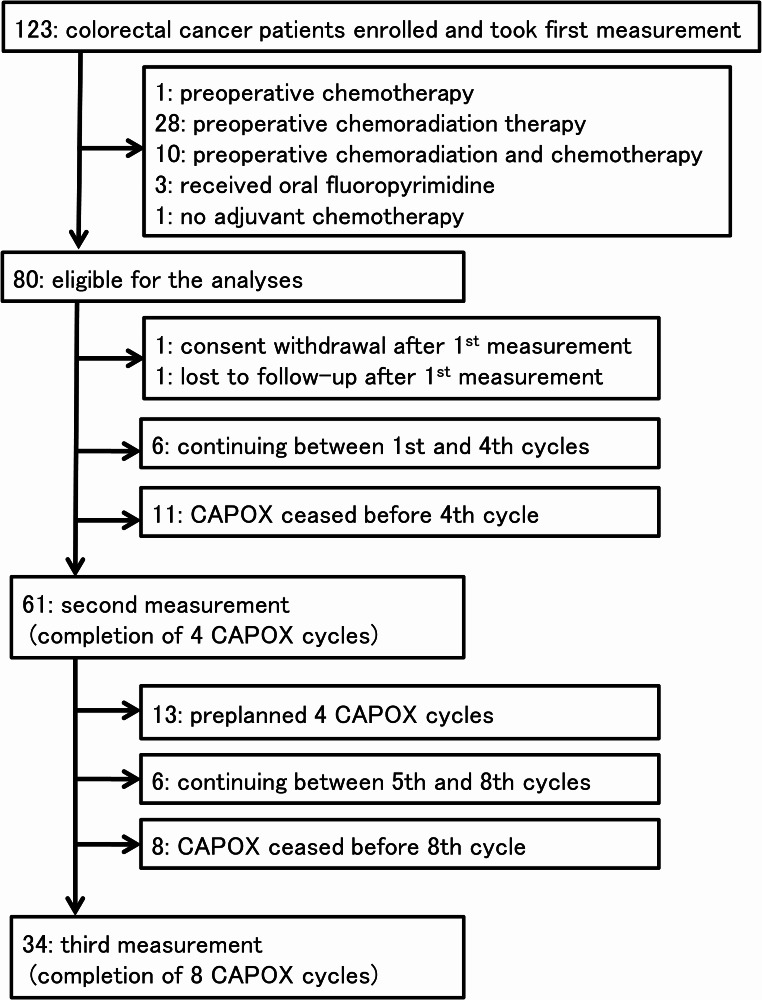
Flow chart to select patients for analyses

Among the 123 patients who initially participated in the present study, 39 who received neoadjuvant therapies and four who did not receive adjuvant CAPOX were excluded. Therefore, 80 participants were initially eligible for analyses (Fig. 2).

The baseline characteristics of 80 patients who underwent the first measurement are shown in Supplemental Table S1. Median age was 61 years, with 46 males (58%). The body mass index was similar between male and female patients. However, women had a higher albumin level and lower hemoglobin level than men. The incidence of well-/moderately differentiated tubular adenocarcinoma (the differentiated carcinoma type) was 88%. The pathological depth of tumor invasion was mostly T3 (49%) or T4 (28%). Only one patient had a stage II tumor.

At the end of May 2024, six patients were still receiving CAPOX between the first and fourth cycles, whereas CAPOX was discontinued before four cycles in eleven. The reason for the early termination of CAPOX was exclusively adverse events. Sixty-one patients underwent the second measurement and completed the four cycles (Fig. 2).

Among the study cohort, four-cycle CAPOX was preplanned in 13 patients. Moreover, six patients were still receiving adjuvant CAPOX between the fifth and eighth cycles. Adjuvant CAPOX was stopped after the second measurement in eight patients, including one male who developed hepatic metastases after the sixth CAPOX cycle and underwent hepatectomy. Therefore, 34 patients underwent the third measurement and completed eight cycles of adjuvant CAPOX, although several were treated with a reduced dosage (Fig. 2).

### Analyses of patients who underwent the second measurement

Supplemental Table S2 shows the details of the first four cycles of adjuvant CAPOX in 61 patients who underwent the second measurement. The dose of capecitabine was slightly lower in women, but there were no sex differences in the dose intensity of oxaliplatin and overall adverse events.

**Fig. 3:**
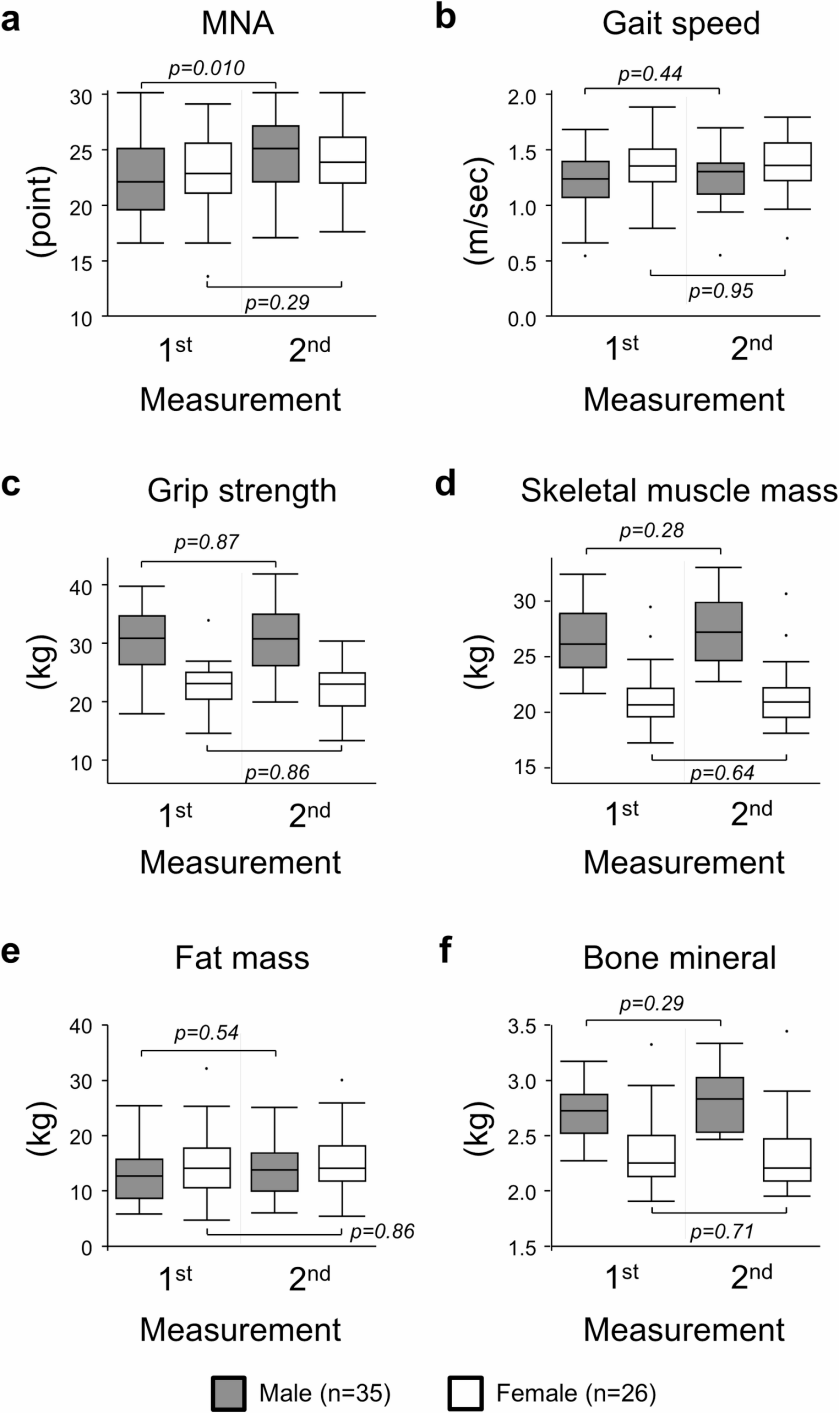
Values of diagnostic metrics of sarcopenia at first and second measurements in patients who completed four cycles of CAPOX. Medians and interquartile ranges are presented for each measurement using box plots (grey boxes: male patients, white boxes: female patients). Differences between measurements within sex were tested using the Wilcoxon rank-sum test. **(a)** Mini Nutritional Assessment (MNA), **(b)** Gait speed, **(c)** Grip strength, **(d)** Skeletal muscle mass, **(e)** Fat mass, and **(f)** Bone mineral content

Figure 3 shows sex-stratified changes in the diagnostic metrics of sarcopenia in this patient group. The values of these metrics were presented in Supplemental Table S3. Median values for grip strength, skeletal muscle mass, and bone mineral content in men were higher, whereas the other metrics did not differ between sexes both the first and second measurements. A significant increase in MNA score was observed in male patients (*p* = 0.010), whereas the increase in gait speed, skeletal muscle mass, fat mass, or bone mineral content did not reach a significance (Fig. 3). In the same cohort, relative changes (the second vs. first measurement) were calculated and compared between men and women (Table 1). In female patients, all the metrics generally remained unchanged after four CAPOX cycles. The median change in skeletal muscle mass or bone mineral content was greater in men than in women in this cohort (+ 2.7% vs. + 1.3%, *p* = 0.021, and + 3.3% vs. 0%, *p* = 0.006, respectively).

**Table 1:** Percentage changes in diagnostic metrics of sarcopenia between the first and second measurements in patients who completed four-cycle treatment

Component	Male (*n* = 35)	Female (*n* = 26)	*p*-value
MNA	+ 10.5% (3.4-22.0%)	+ 2.9% (-2.6-13.3%)	0.067
Gait speed	+ 4.5% (-9.4-17.1%)	-2.6% (-10.0-6.6%)	0.16
Grip strength	+ 1.8% (-4.2-8.0%)	+ 2.8% (-8.1-5.6%)	0.66
Skeletal muscle mass	+ 2.7% (1.2-5.1%)	+ 1.3% (-0.5-3.2%)	0.021
Fat mass	+ 4.5% (-1.2-13.1%)	+ 3.5% (-4.4-12.0%)	0.59
Bone mineral	+ 3.3% (-1.4-5.4%)	0.0% (-1.7-1.4%)	0.006

Data were presented as median percentage changes (interquartile ranges). *P-*values were calculated using the Wilcoxon rank sum test. MNA: Mini Nutritional Assessment

The numbers of sarcopenic patients in this cohort were shown in Supplemental Table S4. The frequency of sarcopenia reduced from 16 to 9% after four cycles of adjuvant CAPOX without obvious sex difference.

### Analyses of patients who underwent the third measurement

Supplemental Table S5 shows the details of eight cycles of adjuvant CAPOX in 34 patients who underwent the third measurement. There were no sex differences in the dose intensity of CAPOX or overall adverse events.

**Fig. 4:**
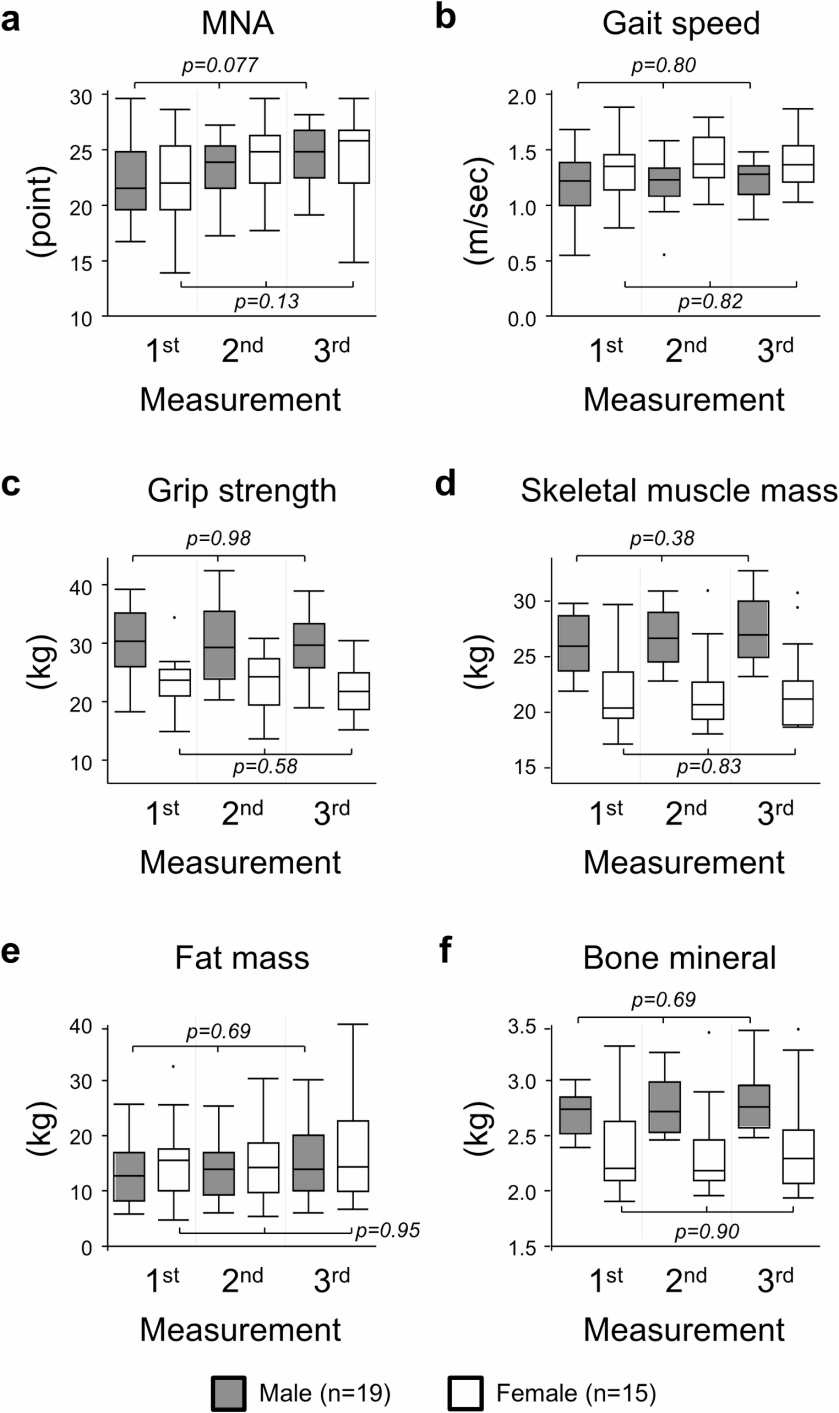
Values of diagnostic metrics of sarcopenia at three measurements in patients who completed eight cycles of CAPOX. Medians and interquartile ranges are presented for each measurement using box plots (grey boxes: male patients, white boxes: female patients). Differences among measurements within sex were tested using the Kruskal-Wallis test. **(a)** Mini Nutritional Assessment (MNA), **(b)** Gait speed, **(c)** Grip strength, **(d)** Skeletal muscle mass, **(e)** Fat mass, and **(f)** Bone mineral content

Figure 4 shows sex-stratified longitudinal changes in the diagnostic metrics of sarcopenia in this patient cohort. The values of these metrics were presented in Supplemental Table S6. Similar to the changes observed between the first and second measurements in the former cohort (*n* = 61, Supplemental Table S3), values for grip strength, skeletal muscle mass, and bone mineral content were higher in men throughout the three measurements, whereas gait speed were faster in women at the second and third measurements. Relative differences in diagnostic metrics between the first and third measurements were calculated and compared between sexes (Table 2). MNA, gait speed, skeletal muscle mass, and fat mass slightly increased in the third measurement from the baseline in male patients. In female patients, MNA, gait speed, and bone mineral slightly increased, whereas grip strength decreased after eight CAPOX cycles. All the above changes over time were not significant, and their differences did not differ between men and women.

**Table 2:** Percentage changes in diagnostic metrics of sarcopenia between measurements in patients who completed eight-cycle treatment

	Second vs. first measurements			Third vs. first measurements		
Component	Male (*n* = 19)	Female (*n* = 15)	*p*-value	Male (*n* = 19)	Female (*n* = 15)	*p*-value
MNA	+ 10.0% (0.0-20.9%)	+ 6.1% (2.3-16.3%)	0.93	+ 8.7% (2.7-15.4%)	+ 8.2% (3.4-22.7%)	0.99
Gait speed	+ 1.1% (-10.8-17.1%)	-0.7% (-6.0-12.2%)	0.99	-1.7% (-4.1-11.3%)	-0.7% (-4.6-19.5%)	0.93
Grip strength	+ 1.7% (-8.0-8.0%)	-0.3% (-7.7-7.1%)	0.99	+ 0.2% (-4.6-6.5%)	-4.1% (-11.3-3.2%)	0.15
Skeletal muscle mass	+ 2.8% (1.2-4.9%)	+ 1.1% (-0.5-4.1%)	0.21	+ 5.9% (1.1-7.3%)	+ 3.9% (-2.0-8.2%)	0.31
Fat mass	+ 2.3% (-1.2-12.4%)	+ 4.0% (-4.0-15.2%)	0.85	+ 12.5% (-5.4-29.8%)	+ 9.8% (-6.5-36.1%)	0.96
Bone mineral	+ 3.3% (-3.2-5.8%)	+ 0.5% (-1.7-2.1%)	0.15	+ 4.0% (-2.1-7.1%)	-0.9% (-3.5-6.8%)	0.54

Data were presented as median percentage changes (interquartile ranges. *P-*values were calculated using the Wilcoxon rank sum test. MNA: Mini Nutritional Assessment

The longitudinal changes in the prevalence of sarcopenia among 34 patients who completed eight-cycle treatment were shown in Supplemental Table S7. The frequency of sarcopenia (16–20%) at the baseline was unchanged after eight cycles of adjuvant CAPOX in male patients, whereas no female patients were found at the second and third measurements; these changes over time and differences between sexes were not significant.

## Discussion

Among the large number of studies conducted on sarcopenia in relation to cancer treatment and/or survival, only a few examined serial changes in body composition in cancer-bearing patients during systemic therapy (Blauwhoff-Bsukemolen et al. 2016; Kurk et al. 2019; Antoun et al. 2019). In contrast, the present prospective study highlighted serial changes in comprehensive diagnostic metrics of sarcopenia in patients who received adjuvant oxaliplatin-based chemotherapy after surgery for CRC. The results obtained herein showed that neither males nor females experienced statistically significant changes in skeletal muscle mass (kg) measured by BIA over four cycles of adjuvant CAPOX therapy. However, its relative change differed significantly between the sexes, with males gaining a median of 2.7% and females gaining a median of 1.3%. The statistical and clinical significance of this sex difference remains unclear.

The loss of skeletal muscle mass is significant after major abdominal surgery due to the physiological impact of surgery, inadequate protein nutrition, and physical inactivity (Farhan et al. 2016). For example, thigh muscle cross-sectional areas decreased by 6.7–9.2% in the first postoperative week after elective abdominal surgery (Vinge et al. 1996; Hardy et al. 2022). Hopkins et al. estimated that the average loss of muscle mass was 0.415% per year in stage I-III CRC patients, whereas long-term observations showed that they gained total adipose tissue at a rate of 7.06%/year (Hopkins et al. 2019). Another study using cancer registries in the Netherlands reported that CRC patients lost 1.9 kg after surgery, but gained 2.9 kg during subsequent chemotherapy (Winkels et al. 2016). A prospective observational study within the CALGB89803 trial comparing adjuvant 5-FU and leucovorin vs. weekly irinotecan plus 5-FU and leucovorin for stage III colon cancer also indicated an increase in body weight during chemotherapy (Meyerhardt et al. 2008). Regarding this sex disparity, the aforementioned study from the Netherlands did not show a relationship between sex and weight change during chemotherapy (Winkels et al. 2016). However, their cohort included patients treated with capecitabine monotherapy and those receiving CAPOX. In addition, they measured body weight after at least six months of adjuvant treatment (Winkels et al. 2016). These differences in metrics and the timing of measurements may have led to conflicting findings between their study and ours.

Bone mineral content did not change over four-cycle CAPOX therapy (relative change: +3.3% in men and 0% in women). Nutritional, hormonal, and treatment factors affect the bone microenvironment. In a rat experiment, 5-FU inhibited bone growth by inducing apoptosis in osteoblasts and chondrocytes through the up-regulation of BAX (Xian et al. 2006). In clinical studies, bone mineral density generally decreased in women with breast cancer following cytotoxic chemotherapy (Hadji et al. 2012; Nisha et al. 2021). On the other hand, chemotherapy did not appear to affect bone mineral density in men with testicular tumors or non-Hodgkin’s lymphoma (Brown et al. 2006). Although the effects of oxaliplatin on bone mineral density have not yet been examined in detail, chemotherapy-induced vulnerability in women may explain the retarded recovery of osseous mineral mass during adjuvant CAPOX.

In contrast to the results obtained in the second measurement, sex differences in relative changes in skeletal muscle mass or bone mineral content were not observed in the third measurement. There are several possible explanations for these distinct results. Only patients with good recovery in these components, regardless of sex, were capable of completing eight CAPOX cycles. Several patients who received reduced doses in later CAPOX cycles may have achieved increases in skeletal muscle and osseous mineral mass before the third measurement because the negative effects of chemotherapy had decreased. Furthermore, the small number of patients evaluated in the third measurement may have led to type II errors in the evaluation of changes in these metrics stratified by sex at this time point.

Although the change was not significant, grip strength was lower in the third measurement than in the first or second measurement in female patients who completed eight CAPOX cycles (Fig. 4; Table 2). Oxaliplatin primarily causes peripheral sensory neuropathy of sensory neurons (Pasetto et al. 2006). In pivotal RCTs comparing adjuvant oxaliplatin-based vs. 5-FU regimens, 64–92% patients developed all-grade neurosensory toxicity during six months of oxaliplatin-including chemotherapy (André et al. 2004; Haller et al. 2011; Land et al. 2007). In contrast, previous findings showed that oxaliplatin did not affect motor neurons (Pasetto et al. 2006). Neuromotor toxicities of all grades were observed in only 7% of patients receiving the FLOX regimen in the NSABP C-07 trial (Haller et al. 2011). On the other hand, Miaskowski et al. conducted a cohort study on chemotherapy-induced neuropathy in cancer survivors and found weaker grip strengths in patients with than in those without neuropathy (Miaskowski et al. 2017). Another recent study demonstrated using electroneurography that oxaliplatin may delay transmission in facial nerves without symptoms (Yigit et al. 2019). Further studies are needed to establish whether accumulating doses of oxaliplatin weaken grip strength.

There was no significant sex difference in the recovery in MNA score among patients who completed adjuvant CAPOX. A previous study showed that elderly CRC patients with an MNA score < 24 were less likely to complete the scheduled cycles of palliative chemotherapy and had a poorer prognosis than well-nourished patients (MNA score ≥ 24) (Aaldriks et al. 2013). On the other hand, a relationship was not observed between MNA and adjuvant chemotherapy cycle in the same study (Aaldriks et al. 2013). In terms of sex, a Spanish cross-sectional study on community dwelling older adults indicated that MNA scores were significantly higher in men than in women (Cuervo et al. 2009). However, sex-specific changes in MNA scores by cancer treatment have not yet been examined.

Men walked a little faster after four cycles of adjuvant CAPOX therapy than at the baseline, while gait speed remained unchanged during the treatment period among women in the present study. However, a broader range of gait speeds may hinder sex differences from reaching a significant level. Therefore, a larger number of patients may be required to clarify whether adjuvant CAPOX has a negative impact on gait in women.

The precision and accuracy of BIA in body composition assessment have been discussed for a long time. Hamilton-James et al. reported that tetrapolar and octopolar BIA devices showed high intra-unit and inter-unit precision with a coefficient variation < 1.5% in repeated measurements of fat mass in 160 healthy Caucasians (Hamilton-James et al. 2021). Compared to reference methods, BIA assessment achieved 3–5% accuracy in studies on healthy individuals (Ceniccola et al. 2019). However, a systematic review of studies using cancer patients or patients undergoing heart or abdominal surgery showed relative differences of 18.8% in total body water, and 15.2% in fat-free mass between BIA and deuterium dilution or dual-energy x-ray absorptiometry (Haverkort et al. 2015), suggesting that muscle mass and bone mineral content could be estimated with similar percent errors. In contrast, CT is reported to estimate body composition with a small measurement error of 1.5–2.5% (Arribas et al. 2022). Moreover, estimated body composition between BIA and reference methods was greater in obese individuals (Deurenberg 1996). The maximum change in body components was 12.5% in the present study (fat mass in men at the third measurement, Table 2) was smaller than the reported range of percent measurement error in BIA. Although the study cohort was comprised mostly of normal-weight subjects, the aforementioned low accuracy of BIA as well as no dietary or urinary restrictions might influence the results in the present study.

There were several other limitations that need to be addressed. This was a single center study with a relatively small sample size. A large percentage of patients did not receive eight cycles of adjuvant CAPOX for a number of reasons, including adverse events and the preplanned schedule of four cycles; they were uniformly excluded in some analyses. Moreover, various doses of CAPOX were administered to the patients included, even in the as-treated analysis. Due to these limitations, statistical power may have been insufficient to reveal potentially distinct results in metrics other than skeletal muscle mass and bone mineral density between the sexes. Furthermore, correlations between the measured metrics and every adverse event were not evaluated. The different levels of serum albumin and hemoglobin at the baseline between sexes may have affected outcomes. Importantly, the observed changes in body composition might not be exclusively caused by CAPOX, since we did not have data of the counterparts followed up without adjuvant chemotherapy. We did not obtain preoperative data, which is another major limitation. Lastly, long-term survival in relation to sequential metrics was not addressed because there was only one case of resectable recurrent disease.

In conclusion, the present results suggest that early cycles of adjuvant CAPOX may have a negative impact on the postoperative recovery of several metrics for diagnosing sarcopenia in female patients. There are few preventive or treatment measures available for the adverse events of 5-FU and oxaliplatin. Based on the present results and the close relationship between chemotherapy tolerability and sarcopenia, physical and/or nutritional interventions may be an alternative method to help patients, particularly women, to complete planned adjuvant CAPOX schedules without drug dose reductions.

## Electronic supplementary material

Below is the link to the electronic supplementary material.

Supplementary Material 1

## Data Availability

The datasets generated and analyzed in the current study are available from the corresponding author on reasonable request.
