# Supplementary material for: Sex differences in recovery from postoperative sarcopenia during adjuvant CAPOX therapy for colorectal cancer
Source: J Cancer Res Clin Oncol. 2024 Oct 26;150(10):478. doi: 10.1007/s00432-024-06013-9 (PMC11512877; doi:10.1007/s00432-024-06013-9)
Supplement: Supplementary file 1 — Supplementary Material 1 [file 432_2024_6013_MOESM1_ESM.docx]

**Supplemental Table 1** Baseline characteristics in all patients evaluated at the first measurement

| Variable | Male  (n = 46) | Female  (n = 34) | *p*-value |
| --- | --- | --- | --- |

| Age, years | 61 (58-68) | 60 (50-68) | 0.12 |
| --- | --- | --- | --- |
| Body mass index, kg/m^2^ | 22.0 (20.4-24.3) | 20.7 (19.2-23.6) | 0.18 |
| Albumin, g/dl | 4.0 (3.9-4.2) | 4.2 (4.0-4.3) | 0.021 |
| Hemoglobin, g/dl | 13.1 (12.4-14.1) | 12.3 (11.5-12.9) | 0.0006 |
| ECOG PS |  |  | N/E |
| 0 | 43 (100%) | 34 (100%) |  |
| ≥1 | 0 (0%) | 0 (0%) |  |
| Comorbid illness |  |  |  |
| Cardiovascular | 18 (39%) | 11 (32%) | 0.53 |
| Pulmonary | 2 (4%) | 3 (9%) | 0.65 |
| Hepatic | 1 (2%) | 1 (3%) | 1.00 |
| Renal | 1 (2%) | 1 (3%) | 1.00 |
| Diabetes mellitus | 10 (22%) | 3 (9%) | 0.14 |
| Autoimmune | 1 (2%) | 2 (6%) | 0.57 |
| Neurological | 1 (2%) | 2 (6%) | 0.57 |
| Psychological/mental disorder | 3 (7%) | 1 (3%) | 0.63 |
| Tumor location |  |  | 0.96 |
| Colon | 31 (68%) | 21 (62%) |  |
| Rectum | 13 (28%) | 11 (32%) |  |
| Colon and rectum | 2 (4%) | 2 (6%) |  |
| Tumor histology |  |  | 0.29 |
| Well- to moderately differentiated | 39 (85%) | 32 (94%) |  |
| Poorly differentiated | 7 (15%) | 2 (6%) |  |
| Pathological TNM classification |  |  |  |
| T stage |  |  | 0.79 |
| T1 | 4 (9%) | 2 (6%) |  |
| T2 | 7 (15%) | 6 (18%) |  |
| T3 | 20 (44%) | 19 (56%) |  |
| T4 | 15 (32%) | 7 (20%) |  |
| N stage |  |  | 0.98 |
| N0 | 1 (2%) | 0 (0%) |  |
| N1 | 31 (57%) | 24 (71%) |  |
| N2 | 14 (31%) | 10 (29%) |  |
| Pathological stage |  |  | 1.00 |
| II (high-risk) | 1 (2%) | 0 (0%) |  |
| III | 45 (98%) | 34 (100%) |  |

Values are presented as numbers of patients (%) or median (interquartile ranges). *P-*values were calculated using Fisher’s exact test, the *χ*^2^ test with Yates correction, or Wilcoxon rank sum test. ECOG PS: Eastern Cooperative Oncology Group Performance Status, N/E: not evaluated.

**Supplemental Table 2** Treatment details of the first four cycles of adjuvant CAPOX in patients who completed four-cycle treatment

| Variable | Male (n = 35) | Female (n = 26) | *p*-value |
| --- | --- | --- | --- |
| Relative dose intensity |  |  |  |
| Capecitabine, % | 96.7 (88.2-100.0) | 92.4 (86.7-97.9) | 0.22 |
| Oxaliplatin, % | 95.4 (86.9-97.7) | 95.8 (82.6.4-97.9) | 0.79 |
| Adverse events |  |  |  |
| Any grade 2 or severer | 34 (97%) | 25 (96%) | 1.00 |
| Any grade 3 or severer | 5 (14%) | 4 (15%) | 0.81 |

Values are presented as numbers of patients (%) or median (interquartile ranges). *P-*values were calculated using Fisher’s exact test, the *χ*^2^ test with Yates correction, or the Wilcoxon rank sum test.

**Supplemental Table 3** Values of diagnostic metrics of sarcopenia at each measurement stratified by sex in patients who completed four-cycle treatment according to sex

| Component | Timing | Male (n=35) | Female (n=26) | *p-*value |
| --- | --- | --- | --- | --- |
| MNA | First measurement | 22.0 (19.8－25.0) | 22.8 (21.0－25.3) | 0.37 |
|  | Second measurement | 25.0 (22.0－26.5) | 23.8 (22.0－25.9) | 0.53 |
| Gait speed (m/sec) | First measurement | 1.24 (1.07－1.39) | 1.35 (1.23－1.49) | 0.075 |
|  | Second measurement | 1.30 (1.12－1.38) | 1.36 (1.23－1.56) | 0.23 |
| Grip strength (kg) | First measurement | 31.1 (26.9－34.6) | 23.5 (21.0－25.2) | <0.0001 |
|  | Second measurement | 31.0 (26.8－34.9) | 23.4 (20.0－25.1) | <0.0001 |
| Skeletal muscle mass (kg) | First measurement | 26.2 (24.5－29.0) | 20.7 (19.6－22.0) | <0.0001 |
|  | Second measurement | 27.3 (24.9－30.0) | 21.0 (19.8－22.1) | <0.0001 |
| Fat mass (kg) | First measurement | 12.6 (8.7－15.7) | 14.0 (10.9－17.4) | 0.37 |
|  | Second measurement | 13.7 (10.0－16.8) | 14.0 (11.8－17.8) | 0.46 |
| Bone mineral (kg) | First measurement | 2.73 (2.56－2.88) | 2.26 (2.15－2.46) | <0.0001 |
|  | Second measurement | 2.84 (2.56－3.03) | 2.21 (2.11－2.46) | <0.0001 |

Data were presented as medians (interquartile ranges). *P-*values were calculated using the Wilcoxon rank sum test. MNA: Mini Nutritional Assessment

**Supplemental Table 4** Prevalence of sarcopenia at each measurement in patients who completed four-cycle treatment

| Timing | Male (n=35) | Female (n=26) | *p*-value |
| --- | --- | --- | --- |
| First measurement | 6 (17%) | 4 (15%) | 1.00 |
| Second measurement | 3 (9%) | 2 (8%) | 1.00 |

Data were presented as numbers of patients (%). *P-*values were calculated using Fisher’s exact test, the *χ*^2^ test with Yates correction, or the Wilcoxon rank sum test.

**Supplemental Table 5** Treatment details of the whole cycles of adjuvant CAPOX in patients who completed eight-cycle treatment and took the third measurement

| Variable | Male (n = 19) | Female (n = 15) | *p*-value |
| --- | --- | --- | --- |
| Relative dose intensity |  |  |  |
| Capecitabine, % | 92.7 (79.5-100.0) | 91.0 (82.6-95.2) | 0.88 |
| Oxaliplatin, % | 89.2 (45.2-96.9) | 80.0 (52.3-97.7) | 0.79 |
| Adverse events |  |  |  |
| Any grade 2 or severer | 19 (100%) | 14 (93%) | 0.44 |
| Any grade 3 or severer | 4 (21%) | 5 (33%) | 0.68 |

Values are presented as medians (interquartile ranges) or numbers (%). *P-*values were calculated using Fisher’s exact test, the *χ*^2^ test with Yates correction, or the Wilcoxon rank sum test.

**Supplemental Table 6** Values of diagnostic metrics of sarcopenia at each measurement stratified by sex in patients who completed eight-cycle treatment according to sex

| Component | Timing | Male (n = 19) | Female (n = 15) | | *p-*value |  |
| --- | --- | --- | --- | --- | --- | --- |
| MNA | | First measurement | 21.5 (19.8－24.8) | 22.0 (19.9－25.0) | 0.74 | |
|  | | Second measurement | 24.0 (21.8－25.3) | 25.0 (22.3－26.5) | 0.34 | |
|  | | Third measurement | 25.0 (22.8－26.5) | 26.0 (22.3－26.8) | 0.68 | |
| Gait speed (m/sec) | | First measurement | 1.22 (1.04－1.36) | 1.35 (1.20－1.43) | 0.15 | |
|  | | Second measurement | 1.23 (1.08－1.32) | 1.37 (1.27－1.50) | 0.032 | |
|  | | Third measurement | 1.28 (1.11－1.35) | 1.37 (1.25－1.53) | 0.046 | |
| Grip strength (kg) | | First measurement | 30.1 (26.0－33.9) | 23.6 (21.3－25.4) | 0.002 | |
|  | | Second measurement | 29.1 (24.0－34.6) | 24.2 (20.3－26.4) | 0.008 | |
|  | | Third measurement | 29.5 (26.6－32.6) | 21.8 (19.0－24.6) | 0.0003 | |
| Skeletal muscle mass (kg) | | First measurement | 25.9 (23.7－28.4) | 20.4 (19.6－22.6) | 0.0004 | |
|  | | Second measurement | 26.6 (24.5－28.8) | 20.7 (19.5－22.4) | 0.0002 | |
|  | | Third measurement | 26.9 (25.0－29.3) | 21.2 (19.1－22.6) | 0.0003 | |
| Fat mass (kg) | | First measurement | 12.6 (8.3－16.4) | 15.4 (10.0－17.4) | 0.64 | |
|  | | Second measurement | 13.7 (9.6－16.8) | 14.1 (10.7－18.3) | 0.69 | |
|  | | Third measurement | 13.7 (10.8－19.5) | 14.2 (10.3－20.8) | 0.79 | |
| Bone mineral (kg) | | First measurement | 2.75 (2.56－2.84) | 2.21 (2.13－2.49) | 0.003 | |
|  | | Second measurement | 2.73 (2.56－2.98) | 2.19 (2.13－2.38) | 0.0004 | |
|  | | Third measurement | 2.77 (2.60－2.91) | 2.30 (2.09－2.53) | 0.001 | |

Data were presented as medians (interquartile ranges). *P-*values were calculated using the Wilcoxon rank sum test. MNA: Mini Nutritional Assessment.

**Supplemental Table 7** Prevalence of sarcopenia at each measurement in patients who completed 8 cycles of CAPOX stratified by sex

| Timing | Male (n=19) | Female (n=15) | *p*-value |
| --- | --- | --- | --- |
| First measurement | 3 (16%) | 3 (20%) | 1.00 |
| Second measurement | 3 (16%) | 0 (0%) | 0.24 |
| Third measurement | 3 (16%) | 0 (0%) | 0.24 |

Data were presented as numbers of patients (%). *P-*values were calculated using Fisher’s exact test.
